# Improvement of Performance for Raman Assisted BOTDR by Analyzing Brillouin Gain Spectrum

**DOI:** 10.3390/s22010116

**Published:** 2021-12-24

**Authors:** Qiang Huang, Junqiang Sun, Wenting Jiao, Li Kai

**Affiliations:** 1 Wuhan National Laboratory for Optoelectronics, School of Optical and Electronic Information, Huazhong University of Science and Technology, Wuhan 430074, China; qiangh0115@hust.edu.cn (Q.H.); wtjiao@hust.edu.cn (W.J.); kaili@hust.edu.cn (L.K.); 2 Hunan Provincial Key Laboratory of Grids Operation and Control on Multi-Power Sources Area, School of Electrical Engineering, Shaoyang University, Shaoyang 422000, China

**Keywords:** BOTDR, spatial resolution, Brillouin gain spectrum analysis, Raman amplification, optical fiber

## Abstract

We propose a simplified partitioned Brillouin gain spectrum (BGS) analysis method to enhance the spatial resolution and measurement accuracy of a Brillouin optical time-domain reflectometer (BOTDR) assisted by a first-order Raman pump. We theoretically derive the mathematical model of the partitioned BGS and analyze the superposition process of sub-Brillouin signals within a theoretical spatial resolution range. We unified all the unknown constant parameters of the calculation process to simplify the partitioned BGS analysis method and the value of the uniform parameter is attained through the system test data and numerical analysis. Moreover, to automate data processing, the starting point of the temperature/strain change is determined by the first occurrence of the maximum Brillouin frequency shift (BFS), then the position where the partitioned BGS analysis method calculation begins is obtained. Using a 100 ns probe pulse and partitioned BGS analysis method, we obtain a spatial resolution of 0.4 m in the 78.45-km-long Raman-assisted BOTDR system, and the measurement accuracy is significantly improved. In addition, we achieve a strain accuracy of 5.6 με and a spatial resolution of 0.4 m in the 28.5-km-long BOTDR without Raman amplification.

## 1. Introduction

Distributed optical fiber sensing has attracted the attention of researchers in recent years due to its unique advantage in realizing multi-point temperature, strain, and other physical measurements. The Brillouin optical time-domain reflectometer (BOTDR) with probe pulse and signal processing equipment connected at the same end has favorable flexibility [1,2], and thus has been widely applied in power cables, oil and gas pipelines, large-scale structure health monitoring [3,4,5,6], etc. However, the performance of the distributed optical fiber sensing system in terms of spatial resolution (SR), measurement accuracy, measurement speed, and maximum sensing distance are mutually restricted. Increasing the width of the probe pulse can attain a better signal-to-noise ratio (SNR), sensing distance, and measurement accuracy of the system, but this method will reduce the SR [7].

To solve the tradeoff between these performance indicators, researchers have proposed various methods. Adopting appropriate pulse coding technology can increase the average power of the probe light and enhance the SR, which is a special practical method to improve the system performance [8,9,10,11]. The advanced data processing method can raise the SNR and SR without increasing the system cost [12,13,14,15]. The iterative subdivision method [14] has been used to improve the SR, where the author exploited the probe pulse with a width of 100 ns to achieve SR of 1.5 m in a 50 km BOTDR. However, this method needs to be combined with energy density distribution (EDD), and thus the complexity of the algorithm is increased. A spatial resolution of 0.3 m was obtained in a 5 m BOTDR using 20 ns probe pulse by analyzing the Brillouin gain spectrum [15], but like the iterative subdivision, the position where the data processing method calculations begin is not given. Raman amplification is an effective approach to prolong sensing distance and realize quasi-transparent transmission [16,17]. The ultra-long sensing distance of 150 km can be accomplished by using coherent detection and Raman amplification [18], but the temperature resolution and SR of the system are 5.2 °C and 50 m, respectively. Meanwhile, by adjusting parameters such as Raman pump power and Erbium-doped optical fiber amplifier (EDFA) gain, the sensing distance of 100 km can be achieved [19], but the temperature accuracy and SR are ±3 °C and 40 m, respectively.

SR is one of the most important performance parameters of the BOTDR, indicating the minimum optical fiber length accurately measured by the system. The experimental spatial resolution is defined as the fiber length of the temperature transition region between 10% and 90% of the peak amplitude [20]. We propose a simplified partitioned analysis method through decomposition of BGS, combined with Raman-assisted amplification, which extends the sensing distance and improves the measurement accuracy and SR of the system. To automate data processing, the beginning calculation point of the proposed method is obtained by analyzing the superposition characteristics of the BGS. We experimentally demonstrate a temperature accuracy of 5.7 °C and a spatial resolution of 0.4 m in the 78.45-km-long Raman-assisted BOTDR system through the partitioned BGS analysis; the sub-meter level SR is thus achieved in the long-distance optical fiber distributed sensing system. Moreover, we verify the effectiveness of a simplified partitioned BGS in a 28.5-km-long BOTDR without the Raman pump.

## 2. Fundamentals of the System

### 2.1. First-Order Raman Assisted BOTDR Theory

We mainly analyze first-order co-directional Raman pumping which maintains the advantage of BOTDR single-ended access. When the incident power exceeds the stimulated Raman scattering (SRS) threshold, stimulated inelastic scattering will occur in some nonlinear medium, which will lead to the pump energy transfer to Raman scattering light. First-order Raman-assisted BOTDR utilizes this nonlinear effect to amplify the probe light and Stokes light (Brillouin scattering light) at ~1550 nm with a higher Raman pump at ~1455 nm, then enhances the SNR of the system and increases the measurement accuracy. In the case of the continuous pump, the coupling equation describing the first-order Raman process is written as follows [21]:

(1) $$\frac{dP_{R}}{dz}=-\alpha_{R}P_{R}-g_{R}\frac{\nu_{R}}{\nu_{s}}P_{R}(P_{s}+P_{B})$$

(2) $$\frac{dP_{s}}{dz}=-\alpha_{s}P_{s}+g_{R}P_{R}P_{s}$$

(3) $$\frac{dP_{B}}{dz}=\alpha_{s}P_{B}-g_{R}P_{R}P_{B}$$

where $P_{R}$, $P_{s}$ and $P_{B}$ are the power of Raman pump, probe light and Stokes light, respectively. $\nu_{R}$ and $\nu_{s}$ are the corresponding frequencies of Raman pump and probe signal. $\alpha_{R}$ and $\alpha_{s}$ are the optical fiber attenuation coefficients corresponding to the wavelengths of the Raman pump and probe pulse, respectively. $g_{R}$ is the gain coefficient of the Raman pump to the probe and Stokes.

### 2.2. Simplified Partitioned BGS Analysis Method

**Figure 1:**
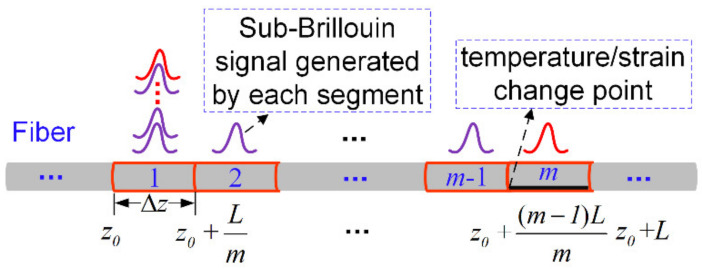
Superposition of sub-Brillouin signal, black section represents the temperature/strain change area. $z_{0}$ is an arbitrary position of optical fiber.

To boost the spatial resolution of the system, we divide the optical fiber into *m* segments within *L*, as shown in Figure 1; the length of each segment is

(4) $$\Delta z=\frac{L}{m}$$

where *L* represents the theoretical spatial resolution of the BOTDR system, which is given by [14]

(5) $$L=\frac{c(\tau +\tau^{\prime })}{2n_{eff}}$$

where *c* is the light speed in vacuum; $n_{eff}$ is the fiber-core effective refractive index; τ is the width of probe pulse; $\tau^{\prime }$ is the response time of the BOTDR detection system, which is determined by the bandwidth of photoelectric devices, such as detectors, filters, and amplifiers [22]. Although the narrow pulse width can improve the spatial resolution of the BOTDR system, it will reduce the frequency resolution and measurement accuracy, which is limited by the phonon lifetime (~10 ns).

As shown in Figure 1, the probe pulse width is set to 100 ns in this experiment, which is greater than the phonon lifetime; the sub-Brillouin signals generated by each segment can thus be approximated by the Lorentzian shape [23]. Hence, the sub-Brillouin signal generated by the *i*th segment is

(6) $$G_{i}(\nu ,\nu_{Bi})=\frac{g_{i}{\left(\Delta \nu_{Bi}/2\right)}^{2}}{{\left(\nu -\nu_{Bi}\right)}^{2}+{\left(\Delta \nu_{Bi}/2\right)}^{2}}$$

where ν is the frequency detuning round the BFS; $g_{i}$, $\nu_{Bi}$ and $\Delta \nu_{Bi}$ are the Brillouin peak gain coefficient, BFS, and the full width at half maximum (FWHM) in the *i*th segment, respectively.

The BGS measured by the system is the overlapping result of sub-Brillouin signals within *L*, thus the BGS at the point $z_{0}$ is obtained as

(7) $$G(\nu ,z_{0})=\sum_{i=1}^{m}a_{i}G_{i}(\nu ,\nu_{Bi})=\sum_{i=1}^{m}a_{i}\frac{g_{i}{\left(\Delta \nu_{Bi}/2\right)}^{2}}{{\left(\nu -\nu_{Bi}\right)}^{2}+{\left(\Delta \nu_{Bi}/2\right)}^{2}}$$

where $G(\nu ,z_{0})$ is the BGS at point $z_{0}$ measured by the BOTDR system; $a_{i}$ represents the constant of proportionality.

Assuming that the temperature/strain change occurs in the *m*th section, as shown in Figure 1, the sub-Brillouin signal of the *m*th can be obtained from (7), which is given by

(8) $$G_{m}(\nu ,\nu_{Bm})=\frac{1}{a_{m}}G(\nu ,z_{0})-\frac{1}{a_{m}}\sum_{i=1}^{m-1}a_{i}\frac{g_{i}{\left(\Delta \nu_{Bi}/2\right)}^{2}}{{\left(\nu -\nu_{Bi}\right)}^{2}+{\left(\Delta \nu_{Bi}/2\right)}^{2}}$$

**Figure 2:**
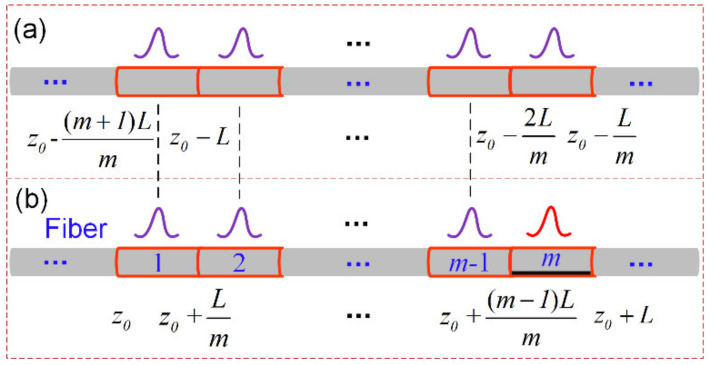
Distribution of sub-Brillouin signal. (**a**) Sub-Brillouin signal within *L* length before z_0_ point. (**b**) Sub-Brillouin signal within *L* length after z_0_ point.

$G(\nu ,z_{0})$ is known, thus the BGS of the *m*th segment can be attained by solving the sub-Brillouin signal from 1st to (*m* − 1)th segment. The sub-Brillouin signal generated by each segment from position $z_{0}$ to $z_{0}+(m-1)L/m$ is approximately equal to that from $z_{0}-(m+1)L/m$ to $z_{0}-2L/m$ because the temperature/strain of these sections have not changed, as shown in Figure 2.

We analyze the sub-Brillouin signal generated within an *L* length before the $z_{0}$ point; the sub-Brillouin signal from $z_{0}-(m+1)L/m$ to $z_{0}-2L/m$ and z_0_ to $z_{0}+(m-1)L/m$ have the same $\nu_{Bi}$ and $\Delta \nu_{Bi}$, which satisfies (9).

(9) $$G(\nu ,z_{0}-\frac{(m+1)L}{m})=\sum_{n=1}^{m}a_{n}G_{n}(\nu ,\nu_{Bn})=\sum_{n=1}^{m}a_{n}\frac{g_{n}{\left(\Delta \nu_{Bn}/2\right)}^{2}}{{\left(\nu -\nu_{Bn}\right)}^{2}+{\left(\Delta \nu_{Bn}/2\right)}^{2}}$$

where $a_{n}$ represents the constant of proportionality.

Using the scan data detected by the BOTDR system from $z_{0}-(m+1)L/m$ to $z_{0}-L/m$ point and performing Lorentzian curve fitting, the BFS and FWHM of the BGS at each point can be obtained. Thus, the sub-Brillouin gain spectrum of the *m*th segment can be calculated if we can attain the gain peak coefficient $g_{n}$. Assuming that $g_{n}$ is equal to 1, the two intermediate variables A and B can then be expressed as

(10) $$A=\sum_{n=1}^{m}\frac{{\left(\Delta \nu_{Bn}/2\right)}^{2}}{{\left(\nu -\nu_{Bn}\right)}^{2}+{\left(\Delta \nu_{Bn}/2\right)}^{2}}$$

(11) $$B=G(\nu ,z_{0}-\frac{(m+1)L}{m})/A$$

From (8), (10), and (11), we can get:

(12) $$G_{m}(\nu ,\nu_{Bm})=\frac{1}{a_{m}}G(\nu ,z_{0})-\frac{FB}{a_{m}}\sum_{i=1}^{m-1}a_{i}\frac{{\left(\Delta \nu_{Bi}/2\right)}^{2}}{{\left(\nu -\nu_{Bi}\right)}^{2}+{\left(\Delta \nu_{Bi}/2\right)}^{2}}$$

where *F* is a constant. We unify all the constants to *k* without affecting the final fitting results of BFS to simplify the calculation, then (12) becomes

(13) $$G_{m}(\nu ,\nu_{Bm})=G(\nu ,z_{0})-kB\times \sum_{i=1}^{m-1}\frac{{\left(\Delta \nu_{Bi}/2\right)}^{2}}{{\left(\nu -\nu_{Bi}\right)}^{2}+{\left(\Delta \nu_{Bi}/2\right)}^{2}}$$

**Figure 3:**
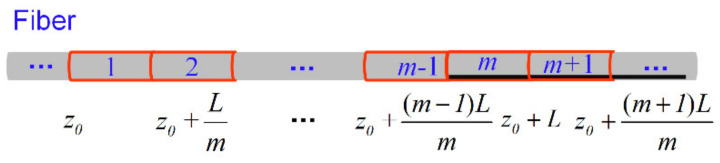
Multi-segment temperature/strain change area. Black indicates the unknown sub-Brillouin signal.

As shown in Figure 3, the BGS generated in the (*m* + 1)th segment can be calculated by (14). By analogy, we can get the sub-Brillouin signal of the following multiple segments.

(14) $$G_{m+1}(\nu ,\nu_{B(m+1)})=G(\nu ,z_{0}+\frac{L}{m})-kB\times \sum_{i=2}^{m-1}\frac{{\left(\Delta \nu_{Bi}/2\right)}^{2}}{{\left(\nu -\nu_{Bi}\right)}^{2}+{\left(\Delta \nu_{Bi}/2\right)}^{2}}-G_{m}(\nu ,\nu_{Bm})$$

**Figure 4:**
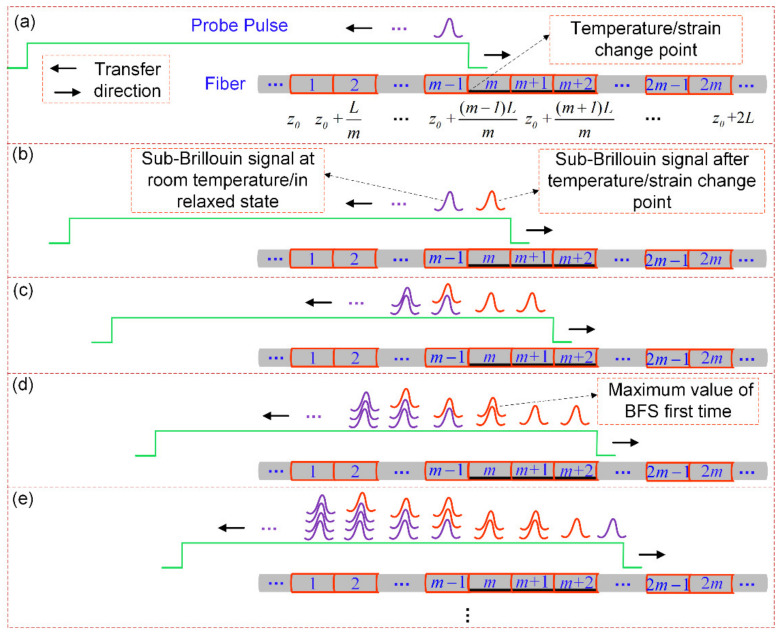
Brillouin gain spectrum superposition process. The black indicates the temperature/strain change area. (**a**) The head of the probe pulse generates a sub-Brillouin signal in the (*m* − 1)th section. (**b**–**e**) is the generation and superposition process of the sub-Brillouin signal of each segment for each *L/m* long distance of the probe pulse propagation, respectively.

Now, the key problem is to find $z_{0}$ in Equation (13), and then the signal processing algorithm can be automated. Assuming that there are three continuous sections where temperature/strain changes, Figure 4 depicts the process of sub-Brillouin signal generation and superposition. In Figure 4b, the sub-Brillouin signal generated in the (*m* − 1)th and *m*th segments after the probe pulse propagates forward for a distance of *L/m*. Since the probe pulse and the Brillouin scattering light propagation are opposite the optical fiber normally, the sub-Brillouin signal generated in the *m*th segment in the previous stage overlaps with the generated in the (*m* − 1)th segment this time, which is shown in Figure 4c. The sub-Brillouin signal completely generated by the temperature/strain sections is superimposed on the *m*th segment (point $z_{0}+(m-1)L/m$) for the first time with the propagation of the light, that is, the BFS appeared as the maximum value. According to the distance between the first BFS maximum point fitted by the system measurement data and the actual temperature/strain starting point *L*, then the following relationship can be obtained:

(15) $$z_{TSC}=z_{0}+\frac{(m-1)L}{m}=z_{MBFS}-L$$

where $z_{TSC}$ is actual the temperature/strain starting point. $z_{MBFS}$ is the first maximum value point of BFS fitted by the system measurement data. The start change point of temperature/strain and $z_{0}$ can thus be obtained using Equation (15).

Combining (15), (13), (14) and its deformations, we can solve the sub-Brillouin signals generated in each segment. Only addition and subtraction are involved in the process of these calculations, thus the partitioned BGS analysis method has no significant effect on the measurement time of the system compared with the process of the data fitting and frequency scanning.

## 3. Experimental Setup and Results

**Figure 5:**
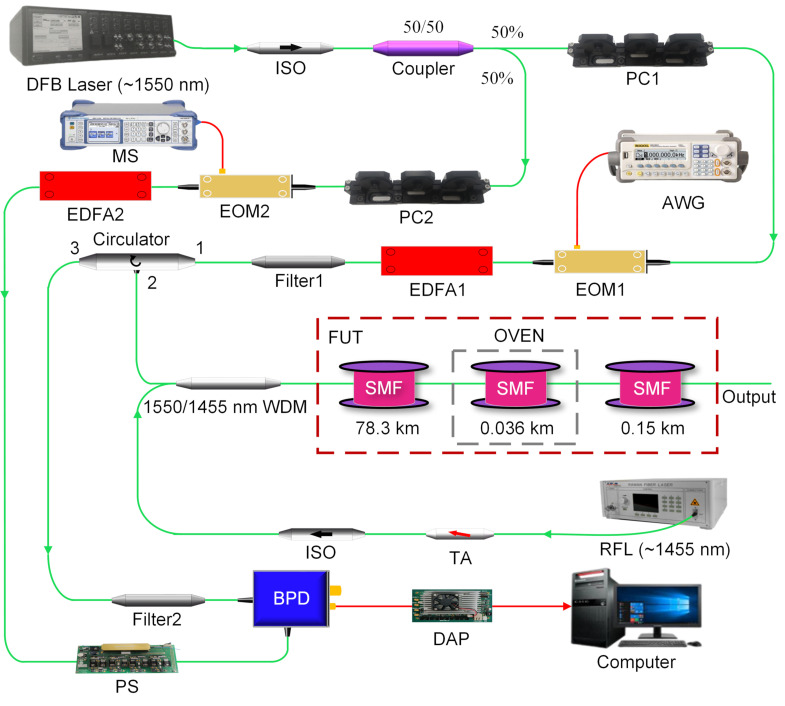
Schematic depiction of Raman-assisted BOTDR system. EOM: electro-optical modulator; WDM: wavelength division multiplexer; TA: tunable attenuator; DFB: distributed feedback; ISO: isolator; PC: polarization controller; BPD: balanced photodetector; EDFA: erbium-doped fiber amplifier; FUT: fiber under test; SMF: single-mode fiber; RFL: Raman fiber laser; PS: polarization scrambler; DAP: data acquisition and processing; AWG: arbitrary waveform generator; MS: microwave source.

**Figure 6:**
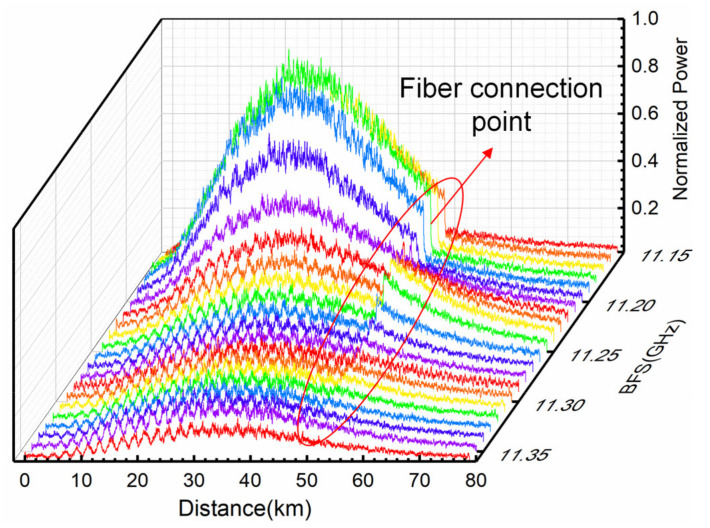
Normalized Brillouin scattering Power–BFS–Distance three-dimensional diagram.

To verify the correctness of the simplified partitioned BGS analysis method, we design the experimental test scheme as shown in Figure 5. The total length of the optical fiber under test (FUT) is 78.45 km, which is composed of two spools with different parameters, with its 36 m section heated and the remaining section kept under room temperature and slack. A distributed feedback (DFB) laser emits continuous-wave light with a wavelength of 1550.074 nm, which is split into the probe and reference light by the 3 dB optical coupler. The probe light passes through the polarization controller (PC), electric-optic modulator (EOM1 bandwidth: 2.5 GHz, extinction ratio ≥25 dB), Erbium-doped fiber amplifier (EDFA1), filter, circulator, and wavelength division multiplexer (WDM), and then enters the FUT. After transiting the EOM2 (bandwidth: 40 GHz, extinction ratio ≥30 dB), EDFA2, and the polarization scrambler (PS) with the 700 MHz scrambling frequency, the reference light beats with backward Brillouin scattering light in the balanced photodetector (BPD) with the 800 MHz bandwidth. A Raman fiber laser (RFL) launches a ~1455 nm Raman pump through a tunable attenuator (TA) and isolator to enter the WDM, then amplifies the probe and scattered light simultaneously. The sampling rate of the data acquisition and processor (DAP) is *f_s_* = 250 MSa/s, and the average times is 1000. The output power of the DFB laser is set to 5 dBm, and the pump power of the RFL after passing the tunable attenuator is 26.43 dBm. Meanwhile, the arbitrary waveform generator (AWG) generates a square wave with a frequency of 1 kHz and a pulse width of 100 ns to modulate the probe light. The ambient temperature is 23 °C, corresponding to the BFS of 11.245 GHz. The temperature of the oven is heated to 80 °C, which corresponds to the BFS of 11.302 GHz according to the temperature correlation coefficient of 1 MHz/°C; in this experiment, and other system parameters such as EDFA pump current are set to the optimal value. The microwave source (MS) frequency scan range is 11.15–11.36 GHz with 10 MHz intervals, and the normalized Brillouin scattering Power–BFS–Distance three-dimensional map is shown in Figure 6 after a five-layer wavelet transforms denoising. In Figure 6, the abrupt drop in power at 50 km is caused by the inconsistent parameters of the two optical fiber spools.

**Figure 7:**
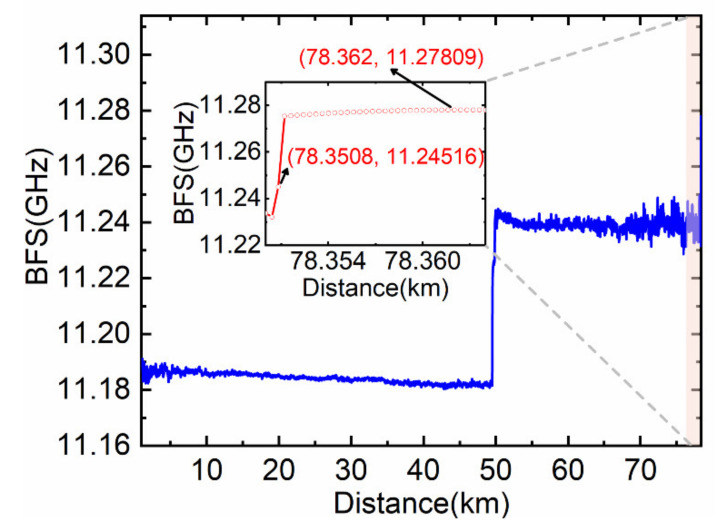
Directly fitting BFS distribution curve.

Direct Lorentzian curve fitting is performed for the measured Brillouin power and the BFS distribution curve along the optical fiber is then obtained, as shown in Figure 7. The fluctuations of the BFS profile are mainly caused by the coiling strain in the fiber, inducing BFS oscillations along the entire sensing range.

It can be seen from the illustration in Figure 7 that the distance from the temperature starting change point (78.3508 km) to the first maximum value of BFS point (78.362 km) equates to the *L* (11.2 m), which is consistent with the result calculated by (15). Moreover, the measured BGS at the heated front end is generated by the superposition of the sub-Brillouin signals from both the unheated and the heated segments, resulting in rather low BFS fitting accuracy.

Since the sampling interval of DAP is *s* = *c*/2*f_s_n_eff_* = 0.4 m, we set Δz *= s* to maintain the continuity of calculation. The optical fiber can then be divided into *m = L/*Δz *=* 28 segments, thus (13) can be transformed into:

(16) $$G_{28}(\nu ,\nu_{B28})=G(\nu ,z_{0})-kB\times \sum_{i=1}^{27}\frac{{\left(\Delta \nu_{Bi}/2\right)}^{2}}{{\left(\nu -\nu_{Bi}\right)}^{2}+{\left(\Delta \nu_{Bi}/2\right)}^{2}}$$

**Figure 8:**
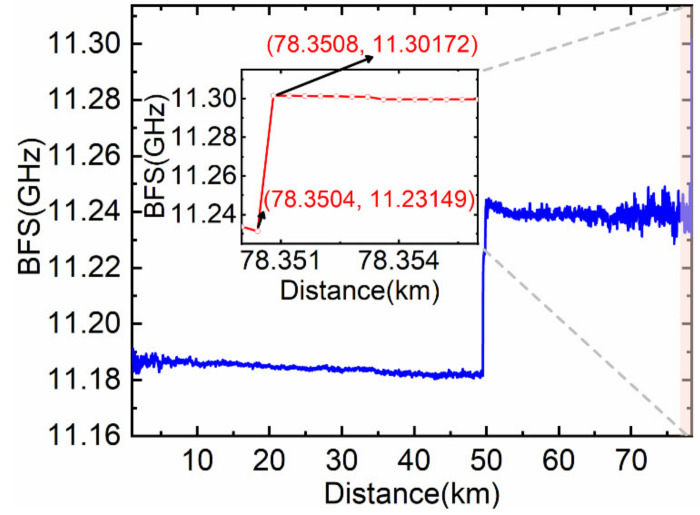
Lorentzian fitting BFS distribution curve after partitioned BGS analysis.

The constant *k* = 0.17 is attained by numerical analysis using known short-distance BOTDR system experimental data. The partitioned BGS analysis method is exploited to calculate the sub-Brillouin signal within the *L* length range (from 78.3508 km to 78.362 km) at the heated front end, and then the BFS distribution in this range is obtained by Lorentzian fitting. Additionally, the BFS distribution beyond this range is obtained by direct fitting to avoid the accumulation of errors and to reduce the calculation time. Finally, the total BFS distribution curve is shown in Figure 8. It can be seen from the illustration in Figure 8 that the spatial resolution and measurement accuracy of the system are improved.

Table 1 shows the comparison between the direct fitting and the fitting result after simplified partitioned spectrum analysis within the *L* length range at the heated front end. As can be seen from Table 1, the temperature accuracy of the direct fitting is 24.2 ℃ in this experiment, obtained from the difference between the measured mean temperature from 78.3508 km to 78.362 km and heating temperature 80℃, which is reduced to 5.7℃ after partitioned BGS analysis, and the spatial resolution is improved to 0.4 m.

**Figure 9:**
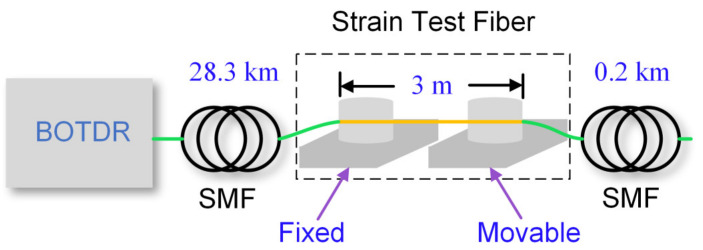
Test scheme of the BOTDR system without Raman pump.

To validate the robustness of the simplified partitioned BGS analysis method in the BOTDR system without the Raman pump and strain sensing, we set up an experimental scheme as shown in Figure 9. A total of 28.5 km length SMF is connected in the BOTDR, and the 3 m section is coiled on two micro-positioners, with one fixed and the other movable for applying uniform strain.

**Figure 10:**
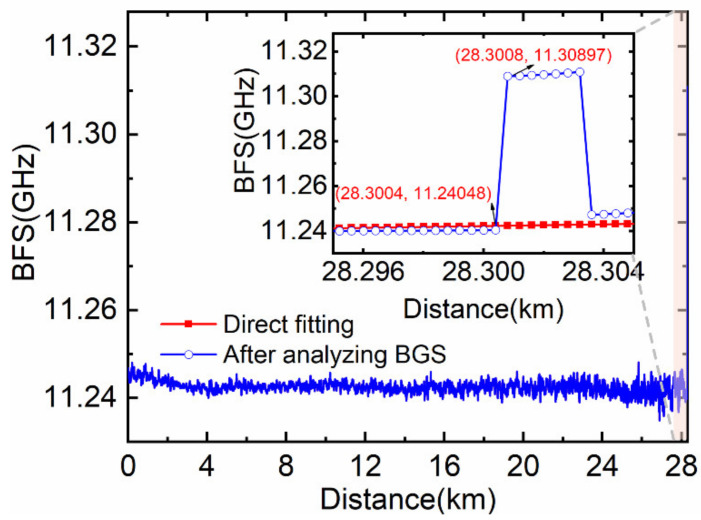
BFS distribution curve of the BOTDR system without Raman pump.

The EDFA pump current and the EOM bias voltage are adjusted to the optimal state, while other system parameters remain unchanged. The axial strain of 1400 με is applied to the 3 m length fiber by moving the precision displacement platform. Then, the BFS distribution curve is obtained by fitting the experimental data shown in Figure 10. Since the measured BGS is a superposed spectrum of the strain and the unstrained section within the spatial resolution and the strained optical fiber length is much less than *L*, the shape of BGS deforms that of the Lorentzian shape. It is obvious in Figure 10 that the results of direct Lorentzian fitting have a significant error and do not reflect the true axial strain in the strain test region. After processing by the partitioned BGS analysis method and then fitting, the mean BFS amplitude of the strain test region is 11.3098 GHz, which is equivalent to the strain of 1405.6 με according to the strain correlation coefficient of the BFS is 4.61 MHz/100με in this experiment. The strain measurement accuracy is 5.6 με, and the rising edge of the BFS distribution curve is 0.4 m, that is, the spatial resolution is 0.4 m. In addition, it can be seen from the blue curve that the applied strain area is 2.8 m, which is close to the actual value of 3 m.

## 4. Conclusions

In summary, we propose an analysis method based on simplified partitioned Brillouin gain spectrum to improve the spatial resolution and the measurement accuracy of the BOTDR system. According to the superposition characteristics of the sub-Brillouin signals, the position where the partitioned BGS analysis method begins to calculate is determined to automate data processing. Moreover, the method of the partitioned BGS analysis is simplified by unifying all the constants to the parameter *k*, which is obtained by the short-distance BOTDR system experiment data and numerical calculation. We set up the first-order co-directional assisted Raman amplification BOTDR system with a 78.45 km length optical fiber to demonstrate the correctness of the proposed method. We utilize the simplified partitioned BGS analysis method to deal with the experimentally obtained Brillouin scattering power in the heated area. The experimental results show that we have achieved a temperature accuracy of 5.7 °C and a spatial resolution of 0.4 m, that is, the sub-meter spatial resolution of the long-distance distributed fiber sensing is realized. Meanwhile, we also designed the strain test scheme of a 28.5-km-long BOTDR system without Raman amplification, in which a 1400 με is applied to strain test fiber with 3 m length. We obtain the spatial resolution of 0.4 m and the strain accuracy of 5.6 με at the far-end fiber by exploiting the proposed method, which is not limited to BOTDR but can also be applied to other fiber optical distribution sensing systems based on Brillouin scattering. Hence, distributed optical fiber temperature/strain sensing based on the method of the partitioned BGS analysis, which has obvious practical application significance, can realize more effective health monitoring for large-scale structures such as pipelines, bridges and power lines.

## Figures and Tables

**Table 1:** Comparison of test results.

Results	Direct Lorentzian Curve Fitting	Lorentzian Fitting after Partitioned BGS Analysis
Mean BFS amplitude (GHz)	11.2778	11.2963
Corresponding temperature (°C)	55.8	74.3
Accuracy (°C)	24.2	5.7
Spatial resolution (m)	11.2	0.4

## Data Availability

Not applicable.
